# From Screening to Therapy: Anti-HCV Screening and Linkage to Care in a Network of General Practitioners and a Private Gastroenterology Practice

**DOI:** 10.3390/pathogens10121570

**Published:** 2021-12-02

**Authors:** David Petroff, Olaf Bätz, Katrin Jedrysiak, Anja Lüllau, Jan Kramer, Hjördis Möller, Renate Heyne, Burkhard Jäger, Thomas Berg, Johannes Wiegand

**Affiliations:** 1 Clinical Trial Centre, University of Leipzig, 04107 Leipzig, Germany; david.petroff@zks.uni-leipzig.de; 2 LADR Laboratory Group Dr. Kramer & Colleagues, 21502 Geesthacht, Germany; o.baetz@ladr.de (O.B.); k.jedrysiak@ladr.de (K.J.); a.luellau@ladr.de (A.L.); j.kramer@ladr.de (J.K.); 3 Leberzentrum am Checkpoint, 10961 Berlin, Germany; hmoeller@leberzentrum-checkpoint.de (H.M.); heyne@leberzentrum-checkpoint.de (R.H.); jaeger@leberzentrum-checkpoint.de (B.J.); 4 Division of Hepatology, Department of Medicine II, Leipzig University Medical Center, 04103 Leipzig, Germany; Thomas.berg@medizin.uni-leipzig.de

**Keywords:** hepatitis C, HCV-RNA, elimination, World Health Organization

## Abstract

(1) Background: Low rates of hepatitis C virus (HCV) diagnosis and sub-optimal linkage to care constitute barriers toward eliminating the infection. In 2012/2013, we showed that HCV screening in primary care detects unknown cases. However, hepatitis C patients may not receive further diagnostics and therapy because they drop out during the referral pathway to secondary care. Thus, we used an existing network of primary care physicians and a practice of gastroenterology to investigate the pathway from screening to therapy. (2) Methods: HCV screening was prospectively included in a routine check-up of primary care physicians who cooperated regularly with a private gastroenterology practice. Anti-HCV-positive patients were referred for further specialized diagnostics and treatment if indicated. (3) Results: Seventeen primary care practices screened 1875 patients. Twelve individuals were anti-HCV-positive (0.6%), six of them reported previous antiviral HCV therapy, and one untreated patient was HCV-RNA-positive (0.05% of the population). None of the 12 anti-HCV-positive cases showed up at the private gastroenterology practice. Further clinical details of the pathway from screening to therapy could not be analyzed. (4) Conclusions: The linkage between primary and secondary care appears to be problematic in the HCV setting even among cooperating partners, but robust conclusions require larger datasets.

## 1. Introduction

In 2016, the World Health Organization approved the strategy to eliminate chronic hepatitis C virus (HCV) infection by the year 2030 [1], which was adopted by the German Ministry of Health with the BIS2030 initiative [2]. In order to succeed, eight key factors are relevant in high income countries: the political will to achieve the WHO elimination targets, a national program that is financed, the implementation/existence of harm reduction programs, the expanded treatment capacity beyond specialists, the removal of treatment restrictions, the monitoring and evaluation of existing programs, the implementation of awareness and screening programs, and the implementation of linkage-to-care programs [3]. In Germany, treatment restrictions for direct antiviral agents do not exist, and modelling data proved the importance of adequate treatment uptake [4], which led to a broad use of modern antiviral therapies in clinical practice. Indeed, nationwide registry data show sustained virological response rates of 97% and excellent tolerability of different treatment regimens [5]. However, improved treatment rates and efficacy are not sufficient to eliminate HCV but should be accompanied by screening and linkage-to-care programs [4]. Thus, we have initiated a hepatitis C screening program on top of a preventive medical examination covered by German health care insurance in the primary care setting and developed screening strategies based on guideline-defined risk scenarios [6]. This approach was successful in identifying previously unknown HCV infections and is in line with the recent national data, which report a higher number of annually diagnosed HCV patients during the years 2018 and 2019 compared to 2017 [7]. However, a follow-up of the index patients from the study by Wolffram et al. indicated that further HCV-specific diagnostics and treatment initiation were sub-optimal because HCV-positive patients usually do not remain at the primary care level but are referred to the secondary care of gastroenterologists/hepatologists or infectious disease specialists [8]. The referral pathways are associated with drop-out rates, and recent Italian data indicated that 39% of the HCV population received antiviral therapy, while only 19% were referred to specialists and 42% were not [9]. Thus, in the present study, we focused on linkage-to-care and used an existing network of primary care physicians and a specialized private practice of gastroenterology to investigate an ideal pathway from the screening to referral of newly identified HCV patients with the documentation of further diagnostic steps and initiation of therapy.

## 2. Patients and Methods

The study was conducted prospectively from September 2019 to October 2020 in Berlin, Germany. All patients provided written informed consent. The study was approved by the Ethics Committees of the University of Leipzig (ethics vote 338/18-ek) and the Medical Association of Berlin (ethics vote Eth-X-1/19). It was registered at the German Clinical Trials Register (DRKS00017704).

The “Check-Up” is a preventive medical examination for adults > 18 years covered by German health care insurance and usually performed by primary care physicians. It covers the patient’s medical history, an evaluation of risk factors, a physical examination, a cholesterol and blood glucose test, a spot urine test, and medical counselling about the results [6]. We additionally analyzed alanine and aspartate aminotransferase (ALT, AST; upper limit of normal 50 U/L for males, 35 U/L for females), gamma-glutamyltransferase (GGT; upper limit of normal 60 U/L for males, 40 U/L for females), and anti-HCV (Cobas, Roche Diagnostics, Mannheim, Germany) in a central laboratory. In all cases in which anti-HCV was positive, HCV-RNA was analyzed by PCR (Cobas Amplicor version 2.0, Roche Diagnostics, Mannheim, Germany, lower limit of detection 15 IU/mL).

The laboratory assessment was combined with 15 questions about risk scenarios for hepatitis C adapted from the German guidelines [10].

Primary care physicians cooperated routinely within an existing network with the secondary care institution of a private gastroenterology practice. Anti-HCV-positive patients were supposed to be referred for further specialized diagnostics and treatment if indicated and at the discretion of the primary care physician. However, the study protocol did not stipulate any details for the referral process and, in particular, no reminders for anti-HCV-positive patients were sent off to the primary care practices so as not to interfere with “real life”.

The primary care physicians received a study-specific honorarium in addition to the “Check-Up” reimbursement of the German health care insurances.

## 3. Statistics

All analyses were performed using the software R version 4.0.2. (Vienna, Austria) Metric variables were compared between groups using a Welch *t*-test and categorical variables with Fisher’s exact test, and the significance level was chosen to be 5%. All other statistics are purely descriptive.

## 4. Results

Within the referral network of the private gastroenterology practice, 237 primary care physicians received a written invitation to take part in the study. The majority did not respond and, of those who did, many declined to participate for lack of time. Finally, 20/237 centers agreed to participate, and 17 (7% of invited primary care physicians) actively recruited 1875 patients within a study period of 14 months (46% male, age 50 ± 17 years). The baseline characteristics of the study cohort are summarized in Table 1.

Twelve individuals were anti-HCV-positive (0.6%), six of them reported previous antiviral HCV therapy but were currently HCV-RNA-negative, whereas one untreated patient was HCV-RNA-positive (0.05% of the population). The remaining five patients were also HCV-RNA-negative. The main risk factor for being anti-HCV-positive was a history of IV drug abuse (5/12 (42%) versus 12/1863 (0.6%); *p* < 0.001). A second statistically significant risk factor was a history of end-stage renal disease with the necessity of hemodialysis (Table 1). Our one HCV-RNA-positive patient answered “yes” to the following risk associated questions: IV drug abuse, tattoo, surgery in the past.

The 12 aforementioned anti-HCV-positive cases arose from five practices, with eight of them coming from a single practice. In four of the twelve cases (from four different practices, aged 29, 40, 43, 72), the physician was not yet aware of the patient being anti-HCV-positive. In one case, the physician performed sonography of the upper abdomen and referred the patient to a specialist outside of the existing network. In the other three cases, the patients were not referred to secondary care.

Thus, further clinical details of the pathway from screening to therapy could not be analyzed.

## 5. Discussion

Although much effort was made to characterize the linkage to care of newly diagnosed hepatitis C patients between primary and secondary care, the project did not proceed as expected and did not allow for a detailed analysis of the patient care pathways. The lack of linkage to care is thus the primary finding.

One reason for the lack of data may be the remarkably low prevalence of anti-HCV and HCV-RNA-positive patients compared to our initial screening project on the primary care level during the years 2012/2013, in which we observed anti-HCV and HCV-RNA prevalences of 0.95% and 0.43%, respectively [6]. Note, however, that the mean age in the current study is 7 years lower and the proportion of IV drug use a factor of seven higher than in the 2012/13 cohort. In the year 2019, the incidence of newly reported hepatitis C cases in Berlin dropped to < 8 cases/100,000 inhabitants compared to 11/100,000 inhabitants during the period 2014–2018 [7]. This may be a consequence of the unrestricted and broad use of direct antiviral agents in real-life with sustained virological response rates of 97% [5].

In November 2020, the Federal Joint Committee (G-BA), the highest decision-making body of the joint self-government of physicians, dentists, hospitals, and health insurance, decided to expand the “Check-Up” examination on the primary care level by including an anti-HCV screening [11]. This decision is the next milestone toward eliminating HCV infection in Germany. However, the linkage to care aspect and the necessity to guarantee a care continuum to successful antiviral therapy will not be affected by this decision. Based on chart audits and physician surveys, successful referral from primary to secondary care occurs in 63% to 82% of patients [12,13,14]. The positive predictors of referral completion are a long relationship between the patient and primary care physician and scheduling the specialty appointment by the primary care physician’s office staff, ideally at the time point the referral is made [15]. In addition, a co-localized setting of primary care and medical doctors treating hepatitis C increases the linkage to subsequent specialists, especially if a health system’s electronic medical record can be leveraged to construct cascades of care [16]. However, in our study, the practices with anti-HCV-positive cases were not directly co-localized to secondary care and reported that their standard practice does not include the direct scheduling of appointments with specialists. Moreover, even in three of the four cases from four practices where the primary care physician and the patient were unaware of an anti-HCV-positive test, the patients were not referred to secondary care. A change to the system in Germany that has recently been implemented is “reflex testing”, meaning that laboratories automatically perform an HCV-RNA PCR test on any blood sample that is anti-HCV-positive and collected within the “Check-Up” program [11]. Another improvement to the system under discussion is the introduction of point-of-care HCV-RNA tests. Currently, the tests available are not necessarily appropriate to the screening setting [17].

Our study was performed within the network of one private gastroenterology practice as a secondary care institution, and we were only able to recruit 7% of the invited primary care physicians of the referral network. Furthermore, the recruitment of patients overlapped with the COVID-19 pandemic, which likely affected the available resources and patient behavior. Thus, our results may not be generalizable to other networks or geographical regions and to the non-pandemic situation.

In conclusion, although much effort was made to characterize the linkage to care of hepatitis C patients between primary and secondary care, the project was unable to collect specific data since a linkage to secondary care did not take place. The prevalence of anti-HCV and HCV-RNA-positive patients was remarkably low compared to our primary screening project and did not allow a more detailed analysis of the patient care pathways.

## Figures and Tables

**Table 1:** Baseline characteristics of the study cohort. Data are means ± standard deviations or numbers (percent). A “limitation” of the results is that “known HCV” is based on a patient report that does not distinguish between currently active or previously resolved HCV infection. Moreover, patient reports may contain inaccuracies.

	Total (n = 1875)	Anti-HCV Negative (n = 1863)	Anti-HCV Positive (n = 12)	*p*-Value
Age (years)	50.4 ± 16.6	50.4 ± 16.7	46.9 ± 11.7	0.32
≤40	639 (34%)	635 (34%)	4 (33%)	1.00
40–60	666 (36%)	659 (35%)	7 (58%)	0.13
>60	570 (30%)	569 (31%)	1 (8%)	0.12
Male	856 (46%)	849 (46%)	7 (58%)	0.40
Elevated ALT	214 (11%)	212 (11%)	2 (17%)	0.64
Elevated AST	99 (5%)	98 (5%)	1 (8%)	0.48
Elevated GGT	241 (13%)	240 (13%)	1 (8%)	1.00
Known elevated liver blood tests	157 (8%)	152 (8%)	5 (42%)	0.0019
Fatigue, impaired concentration, upper abdominal pain	597 (32%)	594 (32%)	3 (25%)	0.76
Medical profession	107 (6%)	107 (6%)	0 (0%)	1.00
Blood transfusion before 1992	91 (5%)	90 (5%)	1 (8%)	0.45
Organ transplantation	16 (1%)	16 (1%)	0 (0%)	1.00
Operation	1157 (62%)	1148 (62%)	9 (75%)	0.39
Piercing	255 (14%)	254 (14%)	1 (8%)	1.00
Tattoo	353 (19%)	348 (19%)	5 (42%)	0.057
IV drug users	17 (1%)	12 (1%)	5 (42%)	<0.001
Known HCV	9 (0%)	7 (0%)	2 (17%)	0.0013
Household member or family with HCV	41 (2%)	41 (2%)	0 (0%)	1.00
HCV therapy in past	16 (1%)	10 (1%)	6 (50%)	<0.001
Serious kidney disease	6 (0%)	5 (0%)	1 (8%)	0.038
You or parents from high risk country	258 (14%)	254 (14%)	4 (33%)	0.070
Travel to high risk country	412 (22%)	410 (22%)	2 (17%)	1.00

## Data Availability

Data can be shared upon individual request.
